# Dynamics of Dissolved and Particulate Polyunsaturated Aldehydes in Mesocosms Inoculated with Different Densities of the Diatom *Skeletonema marinoi*

**DOI:** 10.3390/md9030345

**Published:** 2011-03-11

**Authors:** Charles Vidoudez, Jens Christian Nejstgaard, Hans Henrik Jakobsen, Georg Pohnert

**Affiliations:** 1 Friedrich Schiller University, Institute of Inorganic and Analytical Chemistry, Lessingstr. 8, D-07743 Jena, Germany; E-Mail: cvidoudez@oeb.harvard.edu; 2 Uni Environment, P.O.Box 7810, N-5020 Bergen, Norway; E-Mail: jens.nejstgaard@bio.uib.no; 3 National Environmental Research Institute, Aarhus University, Box. 358, Frederiksborgvej 399, 12 DK-4000, Roskilde, Denmark; E-Mail: hhja@dmu.dk

**Keywords:** chemical defence, plankton blooms, programmed cell death, oxylipins, heptadienal

## Abstract

A survey of the production of polyunsaturated aldehydes (PUA) of manipulated plankton communities is presented here. PUA are phytoplankton-derived metabolites that are proposed to play an important role in chemically mediated plankton interactions. Blooms of different intensities of the diatom *Skeletonema marinoi* were generated in eight mesocosms filled with water from the surrounding fjord by adding different amounts of a starting culture and nutrients. This set-up allowed us to follow PUA production of the plankton community over the entire induced bloom development, and to compare it with the natural levels of PUA. We found that *S. marinoi* is a major source for the particulate PUA 2,4-heptadienal and 2,4-octadienal (defined as PUA released upon wounding of the diatom cells) during the entire bloom development. Just before, and during, the decline of the induced diatom blooms, these PUA were also detected in up to 1 nM concentrations dissolved in the water. In addition, we detected high levels of the PUA 2,4-decadienal that was not produced by the diatom *S. marinoi*. Particulate decadienal correlated well with the cell counts of the prymnesiophyte *Phaeocystis* sp. that also developed in the fertilized mesocosms. Particulate decadienal levels were often even higher than those of diatom-derived PUA, indicating that PUA sources other than diatoms should be considered when it comes to the evaluation of the impact of these metabolites.

## 1. Introduction

Polyunsaturated aldehydes (PUA) are phytoplankton-derived compounds which are common in the world ocean [1,2]. PUA bear an α,β,γ,δ-unsaturated aldehyde structure element and are biosynthetically derived from unsaturated fatty acids [3]. The main class of organisms in the plankton producing PUA are diatoms, and a survey of cultured isolates revealed that *ca*. one-third of all marine diatoms tested are capable of producing these metabolites [4]. Other microalgae have been shown to produce PUA as well. In particular, the prymnesiophyte *Phaeocystis pouchetii* is a source of the PUA decadienal [5]. A recent field survey also suggests that hitherto unidentified picoplankton can be a source of PUA within the plankton [1].

Even after more than ten years of research on the topic, the extent of PUA influence on ecological interactions in the plankton remains a matter of debate [2,6,7]. The PUA 2,4-decadienal and 2,4,7-decatrienal from the diatom *Thalassiosira rotula* have been initially identified as the compounds responsible for impairing the hatching success of the eggs of copepods, that were feeding on diatom blooms in the Adriatic Sea [8]. Other PUA from diatoms, including 2,4-heptadienal and 2,4-octadienal, exhibited inhibitory activity on the hatching of copepod and sea urchin eggs as well [9]. In addition, a multitude of other detrimental effects have been reported for the reactive PUA. Mainly tested in laboratory experiments, these metabolites exhibited toxicity towards a multitude of other herbivores, phytoplankters and microbes (see [10] for a review). Apart from direct and indirect toxic effects, PUA are also suspected to play a role in cell-cell communication. These metabolites can trigger signaling pathways in the diatom *Phaeodactylum tricornutum*, involving Ca^2+^ and NO production [11]. Thus, PUA were speculated to be potential signals involved in synchronized bloom termination events. Supporting this role of PUA, Vidoudez and Pohnert observed that nanomolar concentrations of PUA added to a batch culture of *S. marinoi* can trigger the declining phase [12].

Motivated by these numerous biological activities of PUA, several studies have focused on the impact of external parameters on PUA production. PUA are produced *de novo* from storage lipids upon mechanical disruption of diatom cells [13]. From here on, the PUA released during wounding are termed “particulate PUA” in order to reflect their origin from phytoplankton cells. This wound-activated process was initially assumed to be the only source of PUA, but recently it became clear that intact cells can also release PUA (referred to as “dissolved PUA”) during the late stationary growth phase [12]. The detected dissolved PUA can result from an active release process or from liberation of PUA upon cell lysis. It is now clear that particulate PUA production is highly variable among different diatoms and can even differ substantially between two isolates of one species [4]. In addition, PUA production depends on several external factors, such as nutrient availability and age of the culture [14,15]. This high variability and the influence of external parameters make it difficult to extrapolate the validity of results from lab studies to field situations. A few studies have been undertaken to monitor particulate PUA production during plankton blooms or plankton successions [1,2,16], but until now no comprehensive picture of the particulate and dissolved PUA concentrations during bloom development has been available.

Since laboratory studies are unable to determine the true concentrations and distribution of PUA in the complex marine environment of the plankton, we decided to undertake a survey over entire phytoplankton bloom cycles using marine plankton communities enclosed in mesocosms. We monitored PUA in un-manipulated mesocosms filled with Norwegian Fjord water as well as in fertilized mesocosms. In addition, enrichments with *Skeletonema marinoi* (Sarno&Zingone) were used to trigger artificial blooms of this diatom. *S. marinoi* is widely distributed in the world’s seas and recurring blooms have been reported [17]. This diatom has been extensively used as a model species in ecological and chemical-ecological studies (see e.g., [18,19]). Different isolates of *S. marinoi* have the capability to produce heptadienal, octadienal and octatrienal after wounding, and also to release these metabolites into the water [12,13,20,21]. Here we present data on both particulate and dissolved PUA during a *S. marinoi* bloom and shed light on the complex dependence of PUA production on dominant species and relative composition of the plankton.

## 2. Results and Discussion

### 2.1. Development of Communities in the Different Mesocosms

**Figure 1.**
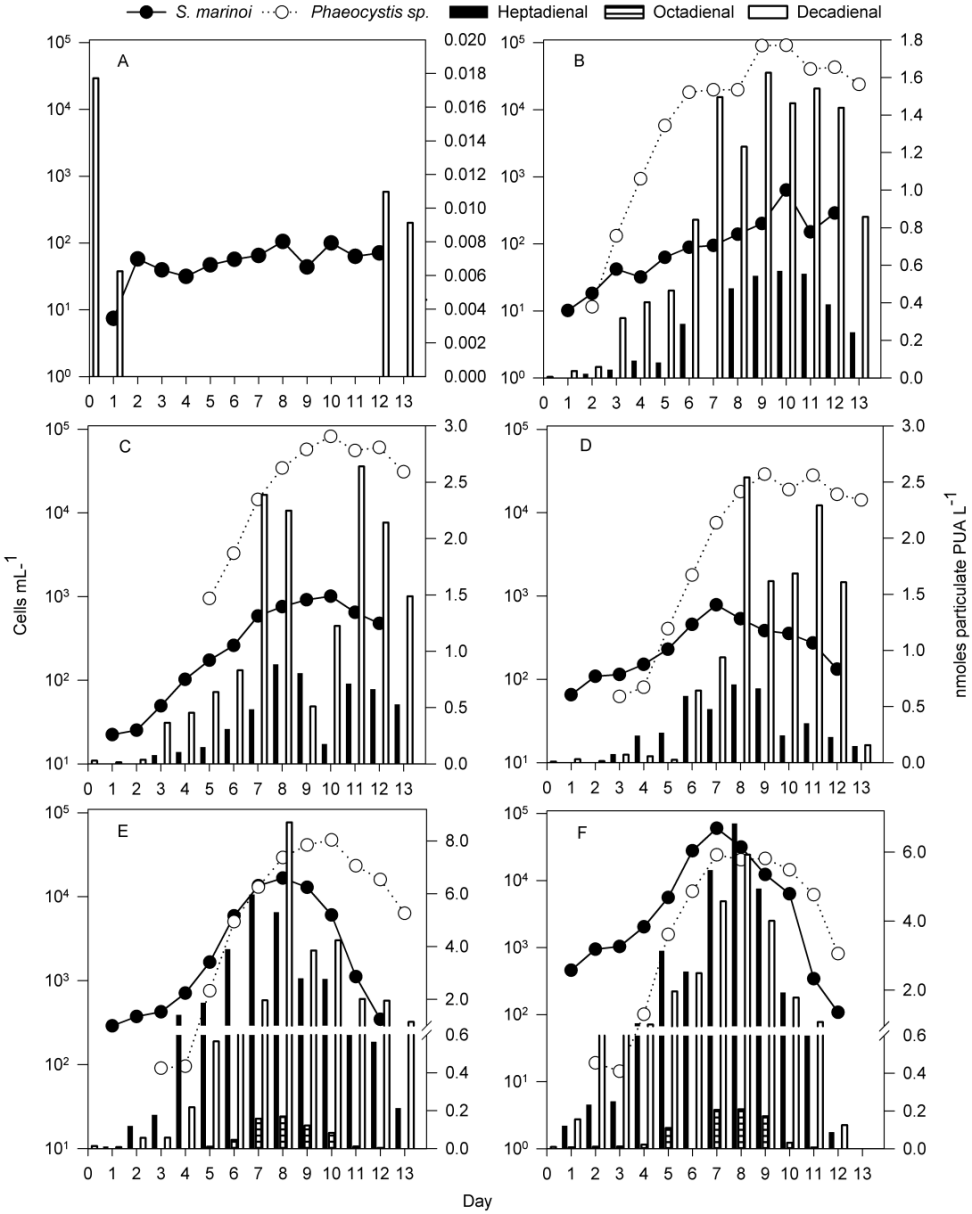
Particulate polyunsaturated aldehydes (PUA) of the cells in one liter of mesocosm water (bars) and cell counts of *S. marinoi* (black dots) and *Phaeocystis* sp. (white circles). (**A**) Mesocosm filled with un-treated fjord water; (**B**) mesocosm filled with fjord water, fertilized with N and P; (**C**) Mesocosm filled with fjord water fertilized with N, P, and Si; (**D**, **E**, **F**) Mesocosms filled with fjord water, fertilized with N, P and Si, and inoculated with *S. marinoi* to reach starting conditions of ∼100 cells mL^−1^, ∼400 cells mL^−1^, and ∼1000 cells mL^−1^, respectively. No relevant amounts of *Phaeocystis* sp. were detected in mesocosm A.

Laboratory experiments have shown that several environmental factors and biotic parameters influence the production and release of particulate and dissolved PUA [12,14]. We undertook mesocosm experiments that allowed us to access and manipulate a natural system, mimicking natural conditions during typical field situations including plankton blooms [22]. In mesocosm A, where fjord water was enclosed without further manipulation, a relatively constant background level of *S. marinoi* was observed that did not exceed 100 cells mL^−1^ (Figure 1A). This mesocosm did not differ from the outside fjord waters in any of the monitored parameters and confirmed earlier studies in this mesocosm set-up that demonstrate that enclosure does not alter the plankton community significantly (data not shown, [22]). When nitrate and phosphate but no silicate were added (mesocosm B), the *S. marinoi* density increased to a maximum of 0.6·10^3^ cells mL^−1^ at day 10 (Figure 1B). When the enclosed natural plankton population was fertilized with nitrate, phosphate and silicate (mesocosm C) the cell densities of *S. marinoi* increased to a maximum of 1000 cells mL^−1^ at day 10 (Figure 1C).

**Figure 2.**
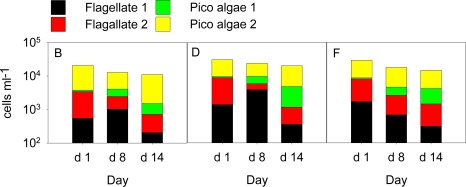
The numerically most important phytoplankters <15 μm in mesocosmos B, D and F at day 1, day 8 and at the end of the experiments determined by the Cytobuoy flow cytometer. The mesocosmos bags C and E developed similarly and the data are therefore not shown.

Inoculation of a fully fertilized mesocosm (D) with 100 cells mL^−1^ *S. marinoi* did not result in a noticeable increase in *S. marinoi* cell count compared to mesocosm C (Figure 1D). In contrast, inoculation with 400 and 1000 *S. marinoi* cells mL^−1^ (mesocosm E and F, respectively) led to a rapid bloom development and maximum densities were reached at day 8 (∼17·10^3^ cells mL^−1^) and day 7 (∼70·10^3^ cells mL^−1^)(Figure 1E and F). In all fertilized mesocosms, we also observed a bloom of *Phaeocystis* sp. with maximum cell counts at day 7–10 (Figure 1B–F). No *Phaeocystis* was detected in mesocosm A. In the mesocosms with nutrients but no induced *S. marinoi* bloom (mesocosms B–D), *Phaeocystis* sp. reached a relatively stable stationary phase with concentrations up to 1 10^5^ cells mL^−1^, while in mesocosms E and F, concentrations started to decline from 5 10^4^ cells mL^−1^ towards the end of the experiment (Figure 1). Blooms of different intensities of the PUA-producing diatom *S. marinoi* could be triggered by manipulating nutrients (nitrate, phosphate and silicate) as well as the cell counts of *S. marinoi* cultures used for inoculation (see also [23]). Since *S. marinoi* was also present in the mesocosms that were not inoculated with the starting culture, we can conclude that this species was a member of the natural plankton community during the field conditions and its abundance was only enhanced in mesocosms B–F. The isolate used for the inoculations was obtained from the Fjord water before and even if we cannot exclude genetic variations we can state that the observed blooms consisted of algae that are native to the environment. Despite the manipulations, we still observed a complex and diverse plankton community in all mesocosms throughout the experiment. Besides the two dominant phytoplankton species discussed here, ciliates as well as heterotrophic and autotrophic dinoflagellates were also detected in varying amounts. These contributed to the overall biomass in the size fraction >15 μm roughly equal to the summed carbon of *Phaeocystis* sp. and *S. marinoi* [23]. Other phytoplankton <15 μm that could potentially also contribute to PUA were observed by flow cytometry (Figure 2). These were coccolithophores, cryptophytes and other un-identified small species. However, none of these cells reached cell abundances near the concentrations observed for *S. marinoi* and *Phaeocystis* sp.. *Phaeocystis* and *S. marinoi* population development scaled well with PUA while the underlying phytoplankton background did not display a similar pattern (Figures 1 and 2). This observation argues in favor of *Phaeocystis* sp. and *S. marinoi* as the main source of PUA. In this mesocosm set-up, we deliberately decided against replicates but rather performed a gradual shift of the natural plankton composition to an induced intense bloom in several steps. This set-up allowed evaluating the contribution of different phytoplankton species to the overall PUA production under different scenarios.

### 2.2. Particulate PUA

Particulate PUA were detected at all time points in the fertilized mesocosms. Analysis of the mass spectra and comparison with authentic standards revealed the presence of three PUA (2,4-heptadienal, 2,4-octadienal and 2,4-decadienal) in variable amounts. Cells from mesocosm A did not produce any particulate heptadienal or octadienal, and decadienal was recorded only at four sampling days (day 0, 1, 12, 13; Figure 1A). In all other mesocosms, all three PUA were detected (Figure 1B–F).

*Heptadienal* and *octadienal -* In mesocosm B, low concentrations of particulate heptadienal were detected (Figure 1B). A similar picture arose in mesocosms C and D, but slightly higher concentrations of these particulate PUA were found (Figure 1C, D). In mesocosm B, particulate heptadienal increased until day 10, which was also the day of the highest *S. marinoi* density. Up to 0.56 nmol heptadienal was produced by the totality of all cells recovered by filtration of 1 L (referred to as nmol cells^−1^ of 1 L from here on)(Figure 1B). In mesocosms C and D, particulate heptadienal reached a maximum at day 8, well in accordance with the *S. marinoi* density. Total concentrations reached 0.88 and 0.69 nmol cells^−1^ of 1 L, respectively (Figure 1C, D). In mesocosm E, where a *S. marinoi* bloom was induced, particulate heptadienal reached 5.96 nmol cells^−1^ of 1 L at day 7, one day before the maximum of *S. marinoi* cell density. In mesocosm F, where an even more pronounced bloom was triggered, up to 6.8 nmol heptadienal cells^−1^ of 1 L were found at day 8, one day after the maximum of *S. marinoi* (Figure 1E and F). If normalized only to the *S. marinoi* cell counts, the particulate heptadienal was similar in all mesocosms (0.3–1.3 fmol *S. marinoi* cell^−1^). This value is in good accordance with PUA production levels of this diatom observed in lab studies [4]. The production rates of total PUA from the strain used for the inoculations in this experiment were varying between 0.2 and 1.7 fmol cell^−1^ dependent on culture conditions in an independent lab experiment. Octadienal was detected only when the *S. marinoi* density was highest, in mesocosm E and F (Figure 1E and F). The particulate concentrations reached a maximum at day 8, measuring 0.17 and 0.21 nmol cells^−1^ of 1 L respectively (10 and 6 attomol if normalized per *S. marinoi* cells). It is interesting to note that in the lab studies higher proportions of octadienal were observed. Since both, the overall level of PUA production as well as the relative ratio of heptadienal:octadienal is dependent on the culture conditions it is possible that a shift towards heptadienal occurs under near natural conditions [14]. The particulate heptadienal was correlated to *S. marinoi* cell density when all mesocosm data were considered and confirmed the connection between this PUA producer and the detected heptadienal (Spearman correlation factor between *S. marinoi* cell density and particulate heptadienal: 0.86, *p* < 0.001; between *Phaeocystis* cell density and particulate heptadienal: 0.66, *p* < 0.001). Best subset regression showed that *Phaeocystis* cell density was not a significant variable in predicting cell potential of heptadienal production (*S. marinoi* cell densities only independent variable, *r*^2^ = 0.59, *p* < 0.001, *S. marinoi* and *Phaeocystis* as independent variables, *r*^2^ = 0.60, *p* < 0.001 for *S. marinoi* variable, *p* = 0.4 for *Phaeocystis* variable). A contribution of the other species in the mesocosm to the overall detected heptadienal and octadienal cannot be fully ruled out. But since the production rates per *S. marinoi* cell match well with lab values and since the other members of the plankton community were not very abundant, we favor the interpretation that 2,4-heptadienal and 2,4-octadienal can be attributed to the induced bloom of the G4 strain of *S. marinoi* that was used for the inoculation.

*Decadienal—*Decadienal was detected in all investigated mesocosms. This PUA is no typical diatom metabolite and, despite numerous surveys, has only been reported once as a natural product from *Thalassiosira rotula* [8]. An alternative source of decadienal could be *Phaeocystis* sp. that always occurred when this PUA was found. In laboratory studies, decadienal was detected from *Phaeocystis pouchetii* from northern Norway water [5]. Indeed it was observed in mesocosm B that the particulate decadienal gradually increased until reaching 0.8–0.9 nmol L^−1^ at day 6. Then, at day 7 when *Phaeocystis* sp. entered stationary phase, particulate decadienal jumped to greater than 1.5 nmol cells^−1^ of 1 L, and stayed relatively stable until day 12. Similar behavior was observed in mesocosms C and D where *S. marinoi* did not bloom (Figure 1C and D). If we assume that *Phaeocystis* sp. is the only decadienal producer, the potential production of PUA would be equivalent to 18–38 attomol decadienal cell^−1^of this algae in mesocosm B. In mesocosms E and F particulate decadienal increased to a maximum at day 8, reaching concentrations of 8.7 and 5.7 nmol cells^−1^ of 1 L, respectively (quantity of this PUA produced by the cells recovered from filtration of 1 L) (Figure 1E and F). The per cell decadienal potential in mesocosms E–F was always higher than in the other mesocosms. If normalized only to *Phaeocystis* sp. cell counts, the detected particulate PUA would correspond to 300 and 290 attomol *Phaeocystis* cell^−1^, respectively.

**Figure 3.**
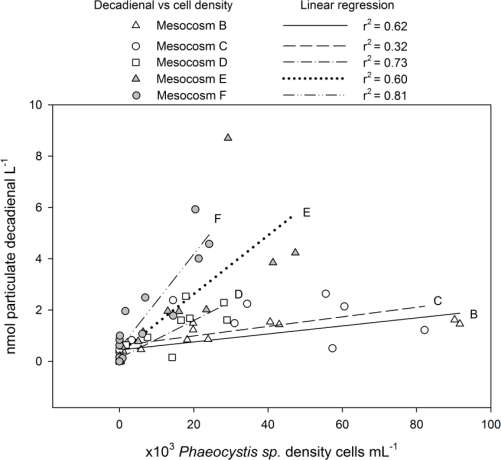
Correlation between particulate decadienal and *Phaeocystis* sp. densities. Letters B–F refer to the mesocosm treatments as described in the legend of Figure 1.

Particulate decadienal was best correlated with *Phaeocystis* cell density if compared to *S. marinoi* cell density, when data from all mesocosms were taken into account (Spearman correlation factor between *Phaeocystis* cell density and decadienal potential: 0.823, *p* < 0.001; between *S. marinoi* cell density and decadienal potential: 0.65, *p* < 0.001). Best subsets regression analysis, however, showed that the regression model was better if *S. marinoi* cell density was also taken into account (*Phaeocystis* as only independent variable, *r*^2^ = 0.117, *p* < 0.001, *Phaeocystis* and *S. marinoi* as independent variables, *r*^2^ = 0.512, *p* < 0.001 for both variables). However, when the data from each mesocosm were considered separately, decadienal correlated with *Phaeocystis* cell density, with influence from *S. marinoi* (Figure 3).

The detection of decadienal in plankton communities requires attention and future experimental work, since it is the most active of the known PUA [9]. The particulate decadienal production of *Phaeocystis* sp. cells increases in all manipulated mesocosms where *S. marinoi* are present in higher proportions (Figure 1C–F). Little is known about the regulation of PUA production in *Phaeocystis*, but it could be hypothesized that an induced PUA production in the presence of a competitor could give a selective advantage to *Phaeocystis* sp. Alternatively, the altered environmental conditions caused by the higher overall cell counts in the mesocosm could result in a non-specific physiological up regulation of the particulate PUA in the prymnesiophyte.

The controlled mesocosm studies presented here allowed us to follow the PUA potential of at least two major bloom forming members of the phytoplankton community that develop in parallel. The activity of PUA depends on the α,β,γ,δ-unsaturated aldehyde structure element found in all detected PUA of this study [9,24]. It can thus be assumed that PUA from different sources can have additive effects if, for example, their toxicity is considered. If only the particulate PUA released upon wounding of the cells are considered, such an additive effect would require feeding of herbivores on both *S. marinoi* and *Phaeocystis* sp. With few exceptions [25] most previous studies focused on the influence of diatom-derived PUA on other members of the plankton community [3,10]. Our survey, however, shows that PUA from other sources, such as *Phaeocystis*, can be dominant in mixed bloom conditions. The fact that apparently other phytoplankton members than diatoms can be the major PUA producers in plankton should be considered if diatom effects are discussed in the light of the action of PUA [26]. This is also supported by our recent survey of a developing *S. marinoi* bloom in the Adriatic Sea that revealed additional PUA-producers with smaller cell sizes compared to the diatom [1].

### 2.3. Dissolved PUA

**Figure 4.**
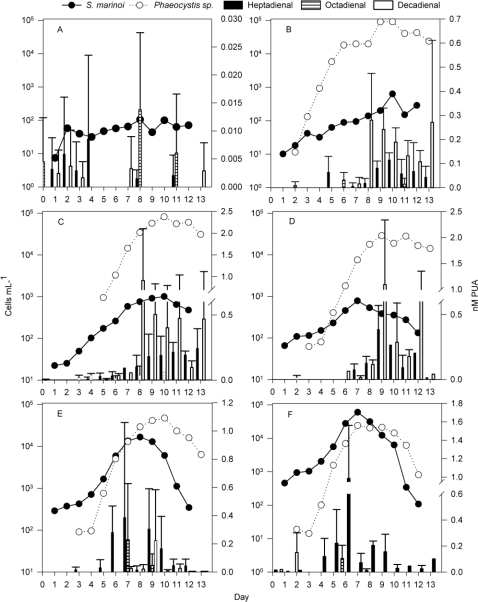
Dissolved PUA (bars), and cell counts of *S. marinoi* (black dots) and *Phaeocystis* sp. (white circles) in mesocosms A–F. PUA values are average ± standard deviation (n = 3). Panels A–F show the mesocosm treatments as described in the legend of Figure 1.

The values of dissolved PUA were very variable, despite that the employed method was previously validated for a unialgal culture of *S. marinoi* [12]. The high variability might arise from interfering biological material in the mesocosms that might absorb partially the released PUA. This is also observed for the internal standard added. The concentration of dissolved PUA was very low (<0.025 nM) or undetectable in mesocosm A (Figure 4A). The dissolved heptadienal concentration remained low in mesocosm B, increasing only up to ∼0.1 nM towards the end of the experiment (Figure 4B). In mesocosms C and D, dissolved heptadienal concentrations increased over the first 8–10 days, reaching comparably high values when *S. marinoi* cell density was starting to slowly decrease (Figure 4C and D). In the mesocosms where *S. marinoi* bloomed, dissolved heptadienal concentrations reached their maximum (0.4 nM and 1 nM for mesocosms E and F, respectively) one day before *S. marinoi* cell densities reached their maxima. In both mesocosms the concentration dropped again on the day of the maximum *S. marinoi* cell density and only subsequently increased again to 0.2–0.3 nM on the first day of the *S. marinoi* decline. The concentrations of heptadienal at the end of the experiments were lower compared to those on day 6–9 (Figure 4E and F). Only very low levels of dissolved octadienal were detected in the mesocosms, without clear trends. In mesocosm B, decadienal was the most abundant dissolved PUA. Its concentration in the water remained very low until day 7 and then increased to reach a concentration of 0.28 ± 0.19 nM at day 8 (Figure 4B). Decadienal concentrations then decreased gradually over the following days with one exception on day 13 (Figure 4B). In the other two mesocosms where nutrients were added but *S. marinoi* did not bloom, the same trend for decadienal was observed (Figure 4C and D). In mesocosms E and F, dissolved decadienal was only found in very low concentrations (Figure 4E and F).

Release of dissolved PUA from *S. marinoi* was previously observed for laboratory cultures. There, PUA release preceded the transition of *S. marinoi* from the stationary to the declining phase [12]. Our mesocosm observations are in good accordance with this release pattern (Figure 4E, F). A role of PUA as signal in stress surveillance of diatoms has previously been proposed and our findings support a possible involvement of released PUA as potential signals [11]. In addition, a delayed second maximum of dissolved PUA was observed in these mesocosms, which was most likely due to the release of PUA during cell lysis of the declining bloom [13]. Dissolved decadienal, however, did not follow such distinct patterns. In mesocosms C and D, where *S. marinoi* only reached low cell counts, dissolved decadienal was clearly detectable. In mesocosms E and F, where *Phaeocystis* cell counts were similar to those in mesocosms C and D, dissolved decadienal was only detected on a few occasions and in low quantities. Further follow-up studies are required to decipher if this is due to absorption or detoxification processes or if the decadienal release is reduced in the presence of the competitor.

## 3. Experimental Section

### 3.1. Mesocosms

Mesocosm experiments were conducted from week 16 to 18 (15–28 April) in 2008 at the Espegrend marine biological field station by the Raunefjord, Western Norway (latitude 60.161°N; longitude: 5.138 °E). For a general description of the location, mesocosm design, filling procedures, and water column mixing see [22]. Six mesocosms (denominated A–F), each 11 m^3^, 2 m diameter, open to air, were filled with 10 m^3^ fjord water from 4 m depth within 5 m of the mesocosm set-up. The transparent polyethylene enclosures were fixed on a frame and kept floating in the surrounding Fjord water. Throughout the experiment mesocosms were kept at natural illumination and bubbled with air. Mesocosm A was kept as a control. At day 1, B was supplemented with 0.4 μM phosphate and 4.24 μM nitrate, while mesocosm C was supplemented with the same concentration of phosphate and nitrate, and 3.61 μM silicate. Mesocosms D–F were supplemented with the same nutrients as C, but in addition, aliquots of a *S. marinoi* culture in exponential phase were added. The diatom culture is maintained at the University of Bergen, Department of Biology (*S. marinoi* strain G4 isolated on 13 June 2006 at the permanent station in the Raunefjord, one nautical mile off the field station, 60°16′N and 5°11′E). The cell density of this diatom reached ∼100 cells mL^−1^ (mesocosm D), ∼400 cells mL^−1^ (mesocosm E) or ∼1000 cells mL^−1^ (mesocosm F) directly after inoculation. Cells were counted using a Cytobuoy™ scanning flow cytometer (CytoBuoy b.v. Woerden, 136 The Netherlands) and a FlowCAM II™ (Fluid Imaging Technologies, Yarmouth, ME, USA) fitted with black and white camera and additionally identified using light microscopy [23].

### 3.2. Sampling

Sampling of mesocosms was performed daily at 9:00 am with 5 L plastic canisters that were transported immediately to the lab. Due to the constant bubbling, the water in the mesocosms was mixed and surface water sampling was sufficient to obtain information about the average situation in the respective mesocosms [22]. Canisters were stored for a maximum of 4 h in a cold room adapted to outside water temperatures until filtration. 0.5–5 L were filtered on GF/C filters under moderate vacuum (*ca.* 500 mbar) for quantification of particulate PUA and 1 L was used for quantification of dissolved PUA as described below.

### 3.3. Apparatus

A GCT premier orthogonal reflectron time-of-flight (oTOF) mass spectrometer (MS) (Waters, Manchester, UK), coupled to an Agilent 6890N gas chromatograph (GC) equipped with a DB-5ms 30 m column (0.25 mm internal diameter, 0.25 μm film thickness, with 10 m Duraguard pre-column, Agilent, Waldbronn, Germany) was used for GC-EI-MS measurements. Helium 5.0 was the carrier gas with a constant flow of 1 mL min^−1^. The electron energy was 70 eV. Samples were injected with a 7683B autosampler (Agilent) equipped with a 10 μL tapered, fixed needle, PTFE-tipped plunger syringe (23–26s/42, Agilent). Calibration of the MS parameters (beam steering, focusing lenses, dynamic range extension (DRE)) was performed just before the analysis of the samples.

### 3.4. Reagents

High grade methanol (Chromasolv © Plus >99.9%, Sigma-Aldrich) and hexane (Suprasolv, Merck, via VWR) were used for all analyses. *O*-(2,3,4,5,6-Pentafluorobenzyl)hydroxylamine hydrochloride (PFBHA, derivatization grade >99%, ABCR, Karlsruhe, Germany) was used for derivatization.

### 3.5. Determination of Particulate PUA

The determination of particulate PUA was done according to the modified protocol described in [1], based on [27]. In short, filtered cells on GF/C filters were suspended in 1 mL of a 25 mM PFBHA solution in Tris-HCl 100 mM, pH 7.2 containing 5 μL of internal standard (benzaldehyde, 1 mM in methanol, Sigma-Aldrich). The cells were disrupted by 1 min of pulsed sonication (0.5 s ultrasound–0.5 s break cycle) to initiate the PUA production. After incubation for 4 h at room temperature the samples were frozen and kept at −20 °C until further analysis. The samples were then extracted with hexane and methanol, and the hexane phase was analyzed by GC-MS.

### 3.6. Determination of Dissolved PUA

Dissolved PUA were quantified using the modified protocol described in [1], based on [12]. In short, three replicate 1 L samples of each mesocosm were extracted on PFBHA preloaded EASY^®^ solid phase extraction cartridges (Macherey-Nagel, Düren, Germany). After elution with methanol, the samples were kept at −20 °C until further analysis. The samples were partitioned with water and hexane, and the hexane phase was analyzed by GC-MS.

### 3.7. Analysis and Quantification of PUA

The analysis and quantification of the PUA was conducted as described in [1]. Statistical analyses were performed with Sigmaplot (version 11.0, Systatsoftwares).

## 4. Conclusions

The multiple roles of PUA in the plankton are yet to be fully understood, but our observation of close-to-natural conditions supports the view of a highly dynamic production and release pattern of these compounds. Besides the influence of overall population composition, the decadienal production that could be attributed to *Phaeocystis* sp. cells is also varying as a function of the density of competitors. Additionally, the dissolved heptadienal concentrations are associated with the growth phase changes of the blooms of *S. marinoi*. These patterns make it likely that PUA production potential and release are either part of signaling pathways and/or a response to stress encountered by the cells.

## References

[1] Vidoudez C, Casotti R, Bastianini M, Pohnert G (2011). Quantification of dissolved and particulate polyunsaturated aldehydes in the Adriatic Sea. Mar Drugs.

[2] Wichard T, Poulet SA, Boulesteix AL, Ledoux JB, Lebreton B, Marchetti J, Pohnert G (2008). Influence of diatoms on copepod reproduction. II. Uncorrelated effects of diatom-derived alpha,beta,gamma,delta-unsaturated aldehydes and polyunsaturated fatty acids on *Calanus helgolandicus* in the field. Prog Oceanogr.

[3] Pohnert G (2005). Diatom/Copepod interactions in plankton: The indirect chemical defense of unicellular algae. ChemBioChem.

[4] Wichard T, Poulet SA, Halsband-Lenk C, Albaina A, Harris R, Liu DY, Pohnert G (2005). Survey of the chemical defence potential of diatoms: Screening of fifty one species for alpha,beta,gamma,delta-unsaturated aldehydes. J Chem Ecol.

[5] Hansen E, Ernstsen A, Eilertsen HC (2004). Isolation and characterisation of a cytotoxic polyunsaturated aldehyde from the marine phytoplankter *Phaeocystis pouchetii* (Hariot) Lagerheim. Toxicology.

[6] Ianora A, Miralto A, Poulet SA, Carotenuto Y, Buttino I, Romano G, Casotti R, Pohnert G, Wichard T, Colucci-D’Amato L, Terrazzano G, Smetacek V (2004). Aldehyde suppression of copepod recruitment in blooms of a ubiquitous planktonic diatom. Nature.

[7] Flynn KJ, Irigoien X (2009). Aldehyde-induced insidious effects cannot be considered as a diatom defence mechanism against copepods. Mar Ecol Prog Ser.

[8] Miralto A, Barone G, Romano G, Poulet SA, Ianora A, Russo L, Buttino I, Mazzarella G, Laabir M, Cabrini M (1999). The insidious effect of diatoms on copepod reproduction. Nature.

[9] Adolph S, Poulet SA, Pohnert G (2003). Synthesis and biological activity of alpha,beta,gamma,delta-unsaturated aldehydes from diatoms. Tetrahedron.

[10] Ianora A, Miralto A (2010). Toxigenic effects of diatoms on grazers, phytoplankton and other microbes: a review. Ecotoxicology.

[11] Vardi A, Formiggini F, Casotti R, De Martino A, Ribalet F, Miralto A, Bowler C (2006). A stress surveillance system based on calcium and nitric oxide in marine diatoms. PLoS Biol.

[12] Vidoudez C, Pohnert G (2008). Growth phase specific release of polyunsaturated aldehydes by the diatom *Skeletonema marinoi*. J Plankton Res.

[13] Pohnert G (2000). Wound-activated chemical defense in unicellular planktonic algae. Angew Chem Int Ed.

[14] Ribalet F, Vidoudez C, Cassin D, Pohnert G, Ianora A, Miralto A, Casotti R (2009). High plasticity in the production of diatom-derived polyunsaturated aldehydes under nutrient limitation: physiological and ecological implications. Protist.

[15] Ribalet F, Wichard T, Pohnert G, Ianora A, Miralto A, Casotti R (2007). Age and nutrient limitation enhance polyunsaturated aldehyde production in marine diatoms. Phytochemistry.

[16] Poulet SA, Escribano R, Hidalgo P, Cueff A, Wichard T, Aguilera V, Vargas CA, Pohnert G (2007). Collapse of *Calanus chilensis* reproduction in a marine environment with high diatom concentration. J Exp Mar Biol Ecol.

[17] Kooistra W, Sarno D, Balzano S, Gu HF, Andersen RA, Zingonea A (2008). Global diversity and biogeography of *Skeletonema* species (Bacillariophyta). Protist.

[18] Yamasaki Y, Nagasoe S, Matsubara T, Shikata T, Shimasaki Y, Oshima Y, Honjo T (2007). Allelopathic interactions between the bacillariophyte *Skeletonema costatum* and the raphidophyte *Heterosigma akashiwo*. Mar Ecol Prog Ser.

[19] Ask J, Reinikainen M, Bamstedt U (2006). Variation in hatching success and egg production of *Eurytemora affinis* (Calanoida, Copepoda) from the Gulf of Bothnia, Baltic Sea, in relation to abundance and clonal differences of diatoms. J Plankton Res.

[20] d’Ippolito G, Romano G, Iadicicco O, Miralto A, Ianora A, Cimino G, Fontana A (2002). New birth-control aldehydes from the marine diatom *Skeletonema costatum*: characterization and biogenesis. Tetrahedron Lett.

[21] Pohnert G, Lumineau O, Cueff A, Adolph S, Cordevant C, Lange M, Poulet S (2002). Are volatile unsaturated aldehydes from diatoms the main line of chemical defence against copepods. Mar Ecol Prog Ser.

[22] Nejstgaard JC, Frischer ME, Verity PG, Anderson JT, Jacobsen A, Zirbel MJ, Larsen A, Martinez-Martinez J, Sazhin AF, Walters T, Bronk DA, Whipple SJ, Borrett SR, Patten BC, Long JD (2006). Plankton development and trophic transfer in seawater enclosures with nutrients and *Phaeocystis pouchetii* added. Mar Ecol Prog Ser.

[23] Barofsky A, Simonelli P, Vidoudez C, Troedsson C, Nejstgaard JC, Jakobsen HH, Pohnert G (2010). Growth phase of the diatom *Skeletonema marinoi* influences the metabolic profile of the cells and the selective feeding of the copepod *Calanus* spp. J Plankton Res.

[24] Adolph S, Bach S, Blondel M, Cueff A, Moreau M, Pohnert G, Poulet SA, Wichard T, Zuccaro A (2004). Cytotoxicity of diatom-derived oxylipins in organisms belonging to different phyla. J Exp Biol.

[25] Hansen E, Even Y, Genevière A-M (2004). The alpha, beta, gamma, delta-unsaturated aldehyde 2-trans-4-trans-decadienal disturbs DNA replication and mitotic events in early sea urchin embryos. Toxicol Sci.

[26] Ianora A, Miralto A (2010). Toxigenic effects of diatoms on grazers, phytoplankton and other microbes: a review. Ecotoxicology.

[27] Wichard T, Poulet SA, Pohnert G (2005). Determination and quantification of alpha,beta,gamma,delta-unsaturated aldehydes as pentafluorobenzyl-oxime derivates in diatom cultures and natural phytoplankton populations: application in marine field studies. J Chrom B.

